# Effects of deprescribing antidepressants in nursing home residents with dementia—a cluster randomized controlled trial

**DOI:** 10.1186/s12875-025-02894-y

**Published:** 2025-06-03

**Authors:** Pernille Hølmkjær, Maarten Pieter Rozing, Gritt Overbeck, Volkert Siersma, Anne Holm

**Affiliations:** Department of Public Health, Center for General Practice, University of Copenhagen, Øster Farimagsgade 5, Building 24, Section Q, 1. Floor, Copenhagen, 1353 Denmark

**Keywords:** Dementia, Deprescribing, Nursing home, Antidepressants, General practice

## Abstract

**Background:**

Older nursing home residents with dementia are commonly prescribed antidepressants despite limited evidence of clinical effect and a high risk of side effects. Deprescribing can be challenging and is often not attempted. The aim of the study is to investigate the effect of a multifaceted intervention targeting nursing home general practitioners and their collaboration with the nursing home staff on the reduction of antidepressant medication in older nursing home residents with dementia.

**Method:**

The study is a cluster-randomized, non-blinded, controlled trial. General practitioners working as nursing home physicians in the Capital Region of Denmark were recruited between June 1 and October 1, 2021. Eligible participants were individuals with dementia (diagnosed or suspected), ≥ 72 years old, receiving one or more antidepressants, and living in a nursing home with the associated nursing home physician**.** The complex intervention consisted of three main parts: 1) a training session occurring in the nursing home, 2) a pre-visit reflection tool, and 3) a dialog tool used during a structured home visit at the nursing home. The control group received enhanced care as usual. Primary outcome was the reduction of the total defined daily dose of antidepressants from pre- to post-intervention in the intervention group, compared to the control group. Secondary outcomes included mortality, changes in other psychotropic medication, hospitalization, and symptoms changes.

**Results:**

We recruited 21 clusters with 128 eligible participants (62/66 in intervention and control). Four clusters withdrew. Most participants were women, and the median age was 85. They received an average of nine different drugs, and the most commonly prescribed antidepressants were sertraline and mirtazapine. The OR for the reduction of antidepressants in the intervention group versus control was 2.3 (95% CI = 0.84–6.2). Mortality rates were similar between groups.

**Conclusions:**

The intervention did not significantly reduce antidepressant use among older nursing home residents with dementia. Further optimization and testing in a larger study are needed.

**Trial registration:**

ClinicalTrials.gov ID NCT04985305, registration date: 2021–08-02.

**Supplementary Information:**

The online version contains supplementary material available at 10.1186/s12875-025-02894-y.

## Introduction

Older nursing home residents with dementia are often treated with antidepressants [1–3]. While treatment with antidepressants in dementia can be indicated, an unknown proportion may not benefit and are at risk of harm due to side effects, including the risk of falls and cardiovascular adverse events [4–8]. Challenges include diagnosing depression and uncertainty about the effectiveness of antidepressants for behavioral and psychological symptoms of dementia (BPSD) [4, 7, 9]. Up to 90% of residents with dementia develop BPSD, whose core symptoms can be indistinguishable from or strongly overlap with symptoms of depression, making a distinction challenging [10–13].

Even though deprescribing in people with dementia is relatively safe, it is rarely initiated [14, 15]. One reason may be the difficulties arising in obtaining sufficient involvement and informed consent in people with cognitive impairment [16, 17]. A deprescribing process requires knowledge of the patient’s medical history, involvement of the general practitioner (GP), and trust, which can be difficult to attain without directly involving the patient [18, 19]. Additionally, relatives and healthcare professionals often express concerns about altering long-term medication due to uncertainty about consequences and doubt about the deprescribing process [20–25].

Most Danish nursing homes are primarily staffed with unskilled laborers, healthcare helpers or healthcare assistants [26, 27]. They maintain the closest contact with the patients, have minimal healthcare training and sparse knowledge of medication and BPSD [28]. However, they often report on changes in behavior including challenging behavior. Their interpretations may contribute to misinterpretation of symptoms, continued use of unnecessary medication, and a reluctance to initiate deprescribing [29–31]. Previous interventions targeting nursing home staff have been effective, but due to a high turnover in staff, the sustainability of such interventions is questionable [29–33]. Thus, we hypothesized that an intervention targeting nursing home GPs with a lower turnover than other healthcare staff, with structured material focusing on knowledge sharing, communication, and involvement, could facilitate sustainable deprescribing [34, 35].

This study aimed to investigate the effect of a multifaceted intervention targeting nursing home GPs and their collaboration with the nursing home staff on the reduction of the total defined daily dose (DDD) of antidepressants from pre- to post-intervention in the intervention, compared to the control group in older nursing home residents with dementia.

## Methods

### Trial design

The study was a pragmatic, non-blinded parallel group cluster randomized controlled trial (cRCT) with 12 months follow-up. This design was chosen due to the type of intervention. The study was registered (NCT04985305), and the protocol was published before completion [34]. We followed the extensions for Consolidated Standards of Reporting Trials (CONSORT) reporting guidelines [36]

### Recruitment and participants

All nursing home GPs in the Capital Region of Denmark were invited to participate. Nursing home GPs in Denmark are organized to cover all or part of a nursing home depending on the size. Patients moving to a nursing home with a nursing home GP may choose to keep their regular GP or transfer to the nursing home GP. Sometimes the nursing home GP thus know the patient from before, or it may be a relatively unknown patient. GPs were recruited by the research team. Patients from each included GP clinic, were eligible if they lived in a nursing home, were ≥ 72 years, received one or more prescriptions of antidepressants and either had a definite diagnosis of dementia from the hospital based on the ICD-10 definition, or were suspected of having a diagnosis of dementia by the GP. The suspected diagnosis was based on the GPs knowledge of the patient and could include a Mini Mental State Examination (MMSE), but it was not mandatory. Exclusion criteria were: receiving end-of-life care, treatment by a psychiatrist, or possible major depressive episode or suicidal behavior. All baseline data was collected by the GPs from their electronic patient database prior to allocation. GPs could recommend inclusion of eligible participants and recorded the inclusion of participants who had moved to the nursing home up to six months after the initiation of the trial.

### Intervention

**Fig. 1:**
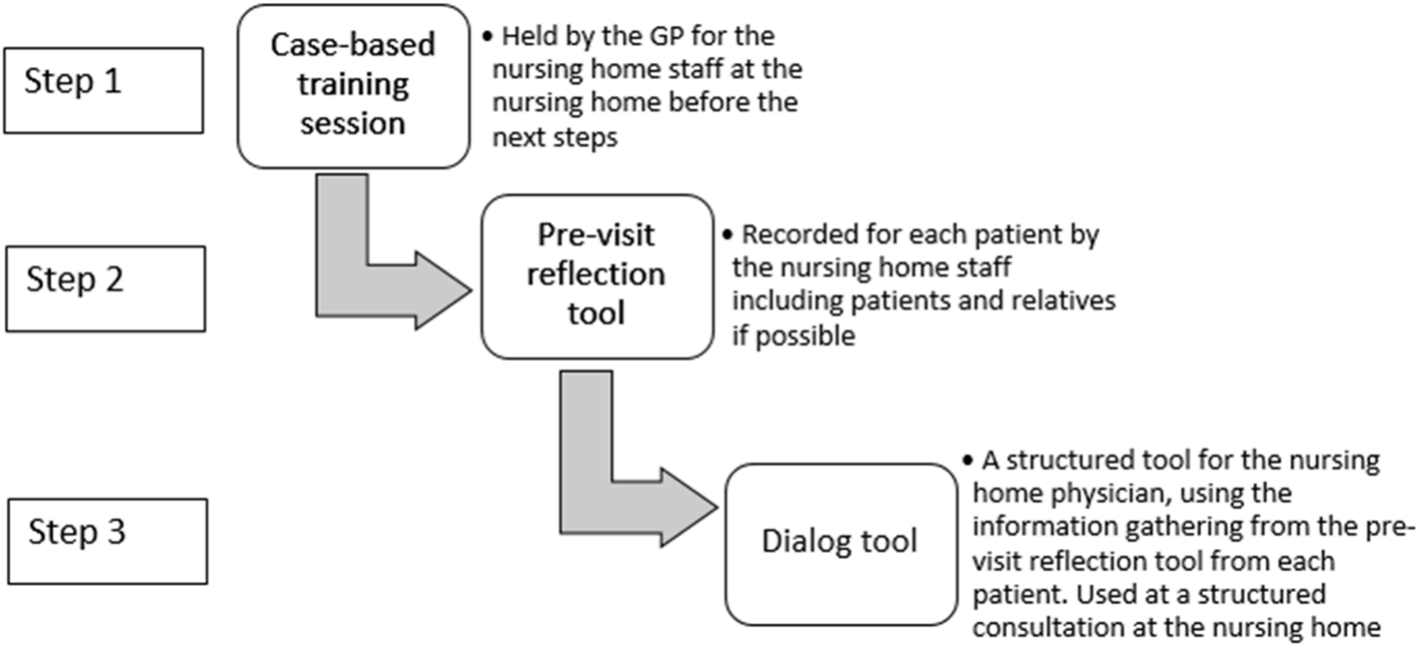
step-wise description on how the three parts of the intervention was used. The three parts of the intervention presented as the three steps were performed. GP = general practitioner

The intervention was developed following the steps from Medical Research Council’s (MRC) framework for developing complex interventions, with additional details presented elsewhere [35, 37]. The intervention consisted of three parts: a case-based training, a pre-visit reflection tool and a dialog tool. The case-based training emphasized the behavioral challenges which may occur with dementia and reasoning for using including how to deprescribe antidepressants. The pre-visit reflection tool was a method to reflect upon the patient’s BPSD symptoms and the staff’s thoughts concerning antidepressants and issues with deprescribing. The dialog tool focused on communication between physician and staff, including their concerns about deprescribing and follow up and was used by the nursing home GP at a structured consultation at the nursing home. First, the case-based training session was held at the nursing home. Second the nursing home staff used the pre-visit reflection tool to record symptoms, thoughts and concerns in a systematic way. Third the dialog tool was used at the structured consultation held at the nursing home with participation of the nursing home GP, nursing home staff and if possible, the patient and their relatives (See Fig. 1). The structured consultation used the information gathered from the pre-visit reflection tool and incorporated nursing home staff and patient/relatives thoughts about deprescribing. Additional information concerning the intervention can be found in the study protocol and the development article [34, 35].

### Trial procedures

Prior to the randomization, all GPs were invited to and participated in a half-day course that included three lectures on non-pharmacological and pharmacological treatment of BPSD, symptoms of dementia, and specific details on procedures for deprescribing. The lectures were provided by experts within the field of treating people with dementia, and pharmacist. Both the intervention and the control group were instructed to have a consultation at the nursing home and fill out a symptom assessment inspired by the Neuropsychiatric Inventory, Nursing Home edition (NPI-NH) before or during the consultation and four weeks after with each participant. The NPI is a self-screening rating tool for the frequency and severity of BPSD in people with dementia. A nursing home edition of the tool (NPI-NH) exists, where nursing home staff rates BPSD and accounts for the staff’s care burden [38, 39].

The intervention and the control group received a visit from the research group, a template for the modified NPI, and a checklist with reminders on the assignments for the trial. The half-day course and the use of NPI constituted the enhanced care-as-usual the control group received. The half-day course did not provide any information not already available to the GPs and it is not always common practice to use the NPI.

The trial lasted from October 14, 2021, to November 30, 2022, during which period the GPs received monthly reminders to print the medical charts of the participating patients. The primary investigator (PH) collected all the data from the GP clinics at trial completion.

### Randomization and blinding

Cluster randomization was performed at the GP clinic level with an allocation ratio of 1:1. A computer-generated algorithm did the randomization and stratified for the size of the clinics to ensure equal distribution of participants. A statistician generated the sequence and assigned the clusters to groups while blinded. The assignment took place after the inclusion of all participants. Due to the type of intervention, it was impossible to blind the GPs and research team after the assignment.

### Outcomes

The primary outcome was any reduction of antidepressants from pre-intervention to the end of the study (12 months). It was measured as a dichotomized response (reduction yes/no) of total antidepressants measured in DDD with data from the electronic medical charts on the day of inclusion and the day of end of study. Thus, if any antidepressant was prescribed on one of these days, they were included in analysis, regardless of the duration of the prescription. Any reduction regardless of the size of the dose was considered a yes. The timing of the primary outcome differed from the protocol, where the outcome measure was three months after the consultation [34]. However, due to a lack of data from the GPs, we did not have medical charts from each month nor a date for when all the consultations took place, thus making it impossible to know when the three months after the consultation occurred. In Denmark, when a patient is dying, it is common practice to stop all medicine and start palliation with 3–4 palliative drugs. Since this is a different process than the deprescribing we tried to introduce and could lead to an overestimation of a possible effect of the intervention, we did not use the medical charts from the end of the study with a clearly end-of-life palliative medicine. Patients who died were included in analyses, using the last available medical chart before the end-of-life medication protocol as the outcome measure. We used the DDD (Defined Daily Dose) to compare and account for the use of multiple antidepressants, including cases where patients switched between medications. This approach considered the varying dosage requirements of different antidepressants. We assessed that any reduction regardless of size was clinically relevant. Secondary outcomes were proportional differences per patient in the total number and each of antidepressants and antipsychotics, anxiolytics, hypnotics, anticonvulsants, analgesics, and anti-dementia medication assessed as changes in DDD. It also included a change in severity in symptom assessment score (modified NPI) four weeks after the structured consultation, the number of hospital admissions within the intervention period due to either falls or any reason, and mortality at the end of the study. All data except from NPI-scores were collected by printing directly from the electronic medical charts from each participant. NPI was recorded by the healthcare staff, kept at the GPs office and collected by PH. All data underwent double entry and checking.

### Statistical analysis

With a sample size based on the primary outcome, we expected a mean reduction of 0.5 and 0.2 antidepressants per person in the intervention and control arm, with an intra-cluster correlation efficiency of 0.10. With a power of 90% and a projected number of 10 patients per cluster, a minimal sample of 182 total (91 in each arm) corresponding to 18 clusters was required. After accounting for anticipated attrition among the clusters of 20%, the adjusted total amounted to 22 clusters.

We described baseline characteristics using median, range, frequency, and percentage. Differences of these baseline characteristics between the randomization groups were assessed by Monte Carlo Wilcoxon tests (continuously valued variables), or Monte Carlo Chi-squared tests (categorically valued variables). A logistic regression model using generalized estimating equations (GEE) was used for the assessment of effects on binary outcomes taking into account clustering for all outcomes. We estimated the odds ratio for the primary outcome as a reduction of total antidepressants, in the intervention group relative to the control group with an unadjusted and an adjusted model. The odds ratio for the secondary outcomes (mortality, each class of antidepressants, each class of other psychotropic medication, hospitalization and change in modified NPI) was estimated the same way. All analyses were conducted intention-to-treat. We adjusted for the predefined baseline variables: years of practice per GP, time the participant had lived in a nursing home, the severity of dementia, the total amount of prescriptions per participant, time on a current antidepressant, receiving antipsychotic or hypnotic or sedative. Multiple imputation was used for missing data. We considered a p-value of < 0.05 significant. All analysis was carried out in SAS v. 9.4.

### Ethics and informed consent

According to the Danish Act on Research Ethics of Research Projects Section "2", the project did not constitute a health research project but was considered a quality development project [40]. A quality development project does not require written informed consent from the participants, but the GPs obtained oral consent to use the patient’s data when possible from either the patient, the legal guardian or a relative. The oral consent was recorded in the medical record for the patient at the GP system. Consent could always be withdrawn. The ethical committee waived the need for ethics approval (Journal no: H-20084023). We obtained written informed consent from participating GPs.

## Results

### Participants

**Fig. 2:**
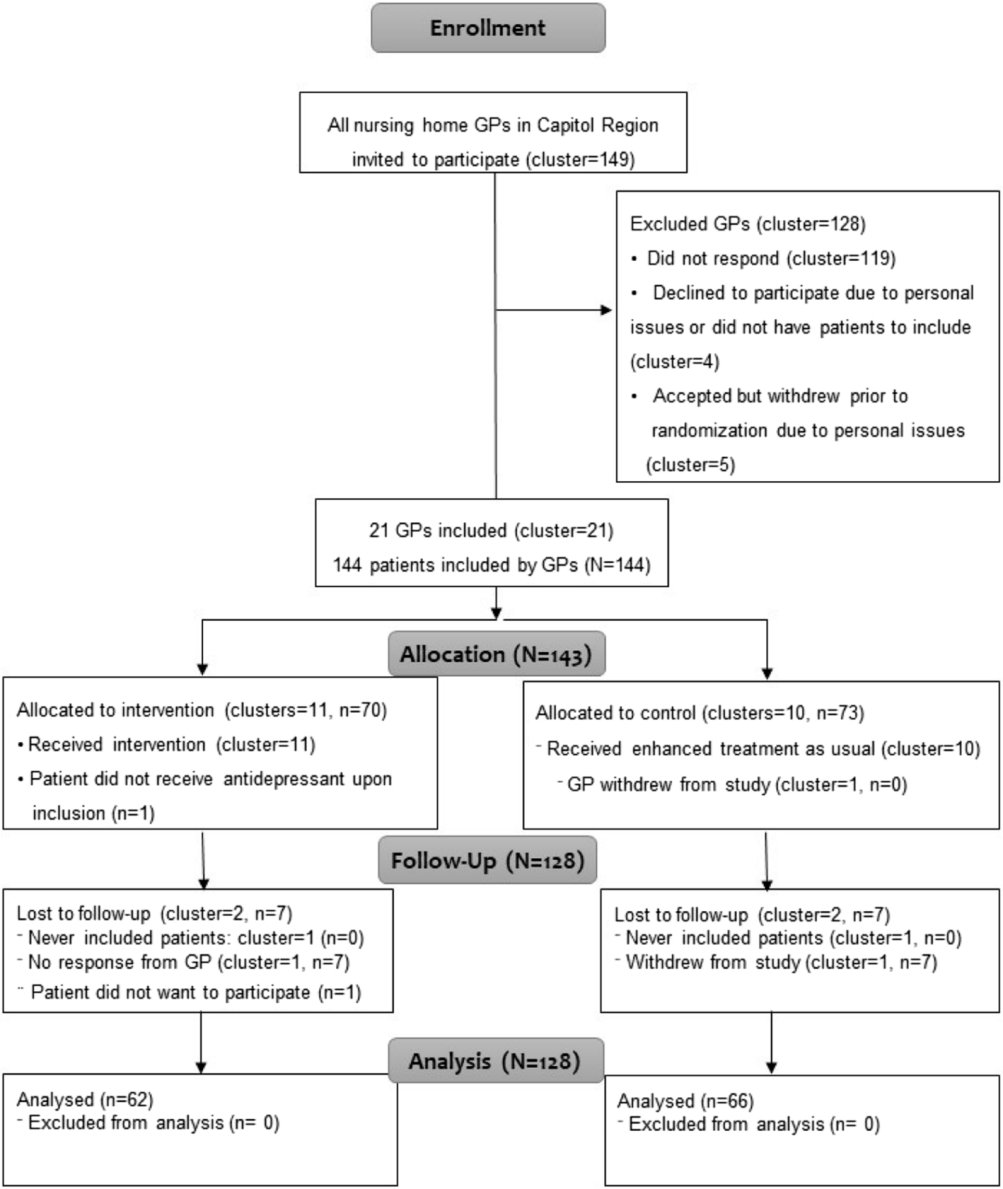
Consolidated Standards of Reporting Trials (CONSORT) Diagram of Participants in the Study. Participant flow and numbers included in primary outcome analysis; CONSORT 2010 flow diagram. CONSORT: Consolidated Standards of Reporting Standards; *N* = all participants, n: sample, GP = general practitioner

Between June 1 st and October 1 st, 2021, we invited 149 GPs to participate (Fig. 2). Of these, 21 GPs were recruited, amounting to 144 eligible participants. Baseline data was collected for all participants. Before randomization, one GP withdrew without having recruited any participants. Four GPs withdrew after randomization (two from each group). Two had not included participants, and two had each recruited seven. One participant withdrew after inclusion. Data from 128 participants was available for analysis on the primary outcome at the end of the trial.

Table 1 shows demographic and baseline data of the study participants. 128 patients were distributed as 62/66 in intervention and control. The two clusters were similar according to the size of the GP clinic and monthly visits from the GP. The GPs in the control group had more years of experience (22 vs. 12), more patients at the nursing homes (55 vs. 38), and more days of sick leave from the staff (16 vs. 10) compared to the intervention group. Almost half of the participants had lived at the nursing home for more than three years and had received current antidepressants for a median of 3 years. In the control group, the participants were slightly younger (85.5 vs. 87.0 years), a higher proportion had a confirmed diagnosis of dementia (73% vs. 45%), there were more prescriptions of selective serotonin reuptake inhibitors (SSRI; 36 vs. 30 prescriptions) and fewer prescriptions of noradrenergic and specific serotonergic antidepressant (NaSSA; 27 vs. 34). Differences were statistically significant for confirmed diagnosis of dementia (*p* = 0.003), antidementia medication (*p* = 0.02) and for unique (*p* = 0.02) and total ATC-codes (*p* = 0.03).

**Table 1:** Baseline data

Clusters (*N* = 16)			
	**Intervention (*n* = 9)**	**Control (*n* = 7)**	***P*-value**^**a**^
Number of patients pr. GP pr. practice (median/range)	1600 (1100–2550)	1500 (875–2400)	0.7347
Years of practice as GP (median/range)	12 (7–30)	22 (4–45)	0.3021
Number of patients living in nursing home (median/range)	60 (40–120)	80 (36–170)	0.3960
Number of patients the nursing home physician attend at each nursing home (median/range)	38 (30–57)	55 (24–150)	0.1251
Monthly visits from GP (number of clusters/%)			0.8489
< 3	1 (11)	1 (14)	
> 3	8 (89)	6 (86)	
Monthly visits from GPs health staff (number of clusters/%)			0.8385
< 1	6 (67)	5 (71)	
> 1	3 (33)	2 (29)	
Sick days per nursing home staff per year (median/range)	10 (1–27)	16 (6–21)	0.1875
Participants (*N* = 128)			
	**Intervention (*n* = 62)**	**Control (*n* = 66)**	
Sex, female (no., (%))	49 (79)	47 (71)	0.4061
Age (median/range)	87 (72–98)	85.5 (73–97)	0.8768
Time lived at nursing home (no., (%))	*(missing:1)*	*(missing:3)*	0.8621
0–3 years	35 (57)	35 (56)	
> 3 years	26 (43)	28 (44)	
Time GP has been nursing home physician for patient (no., (%))	*(missing:6)*	*(missing:3)*	0.6671
0–2 years	23 (41)	30 (48)	
2–5 years	28 (50)	26 (41)	
> 5 years	5 (9)	7 (11)	
Confirmed diagnosis of dementia (no., (%))	26 (45) *(missing:4)*	48 (73)	0.0025
Type of dementia for participants diagnosed, (no., (%))		*(missing 1)*	0.7506
Alzheimer’s	17 (65)	29 (62)	
Vascular	3 (12)	7 (15)	
Lewy-body	2 (8)	1 (2)	
Parkinson’s with dementia	2 (8)	3 (6)	
Other	2 (8)	7 (15)	
Severity of dementia (no., (%))	*(missing: 10)*	*(missing: 18)*	0.8967^b^
Mild	10 (18.9)	8 (16.8)	
*Confirmed diagnosis*	*0 (0.0)*	*4 (50)*	
*Suspected diagnosis*	*10 (100)*	*4 (50)*	
Moderate	29 (54.7)	26 (54.2)	
*Confirmed diagnosis*	*13 (44.8)*	*19 (73.1)*	
*Suspected diagnosis*	*16 (55.2)*	*7 (26.9)*	
Severe	13 (25.0)	14 (29.2)	
*Confirmed diagnosis*	*10 (76.9)*	*14 (100)*	
*Suspected diagnosis*	*3 (23.1)*	*0 (0.0)*	
Number of unique ATC-codes pr. participant^c^ (median/range)	4 (1–8)	5 (1–9)	0.0160
Number of total medications based on ATC-codes pr. participant^d^ (median/range)	8 (2–17)	9.5 (4–18)	0.1219
*Repeat*	*6.5 (1–16)*	*8 (1–14)*	0.0305
*PRN*	*2 (0–7)*	*2 (0–6)*	0.8788
Years participants received current antidepressant (median/range)	3 (0–22) *(missing: 15)*	3 (0–16) *(missing: 7)*	0.9037
Number of prescriptions			
	**Intervention (*n*** **= 62)**	**Control (*n*** **= 66)**	
Number of prescriptions for different classes of antidepressants^e^			
SSRI (N06 AB…)	30	36	0.5898
NaSSA (N06 AX11 + N06 AX03)	34	27	0.1597
SNRI (N06 AX16 + N06 AX21)	6	7	>.9999
TCA (N06 AA…)	1	1	>.9999
Melatonin agonist (N06 AX22)	1	1	>.9999
Serotonin-modulating drugs (N06 AX26)	0	1	>.9999
Number of prescriptions for each drug class^f^			
Antidementia (N06D)	14	29	0.0154
Anticonvulsants (N03)	7	3	0.1925
*Repeat*	7	3	0.1925
Analgesics (N02)	57	64	0.2617
*Repeat*	*28*	*33*	*0.6053*
Antipsychotics (N05 A)	9	8	0.8041
*Repeat*	*7*	*7*	> *.9999*
Anxiolytic (N05B)	10	13	0.6613
*Repeat*	*5*	*5*	> *.9999*
Hypnotic or sedatives (N05 C)	11	17	0.2943
*Repeat*	*10*	*13*	*0.6509*

Baseline data for the study population

*GP* General Practitioner, *ATC* Anatomical Therapeutic Chemical, *SSRI* Selective Serotonin Reuptake Inhibitor, *NaSSA* noradrenergic and specific serotonergic antidepressant, *SNRI* Serotonin and norepinephrine reuptake inhibitors, *TCA* Tricyclic antidepressant

^a^*p*-value of a Monte Carlo Wilcoxon test (continuously valued variales), or a Monte Carlo Chi-squared test (categorically valued variables)

^b^Test of the distribution of dementia severity between the randomization groups; disregarding whether the diagnosis was confirmed or suspected

^c^Excluding antidepressant medication

^d^Some participants received more than one antidepressant

^e^Defined as all ATC codes per participant

^f^Some participants received both Pro re nata and repeat prescriptions of a drug class

128 participants had complete follow-up data and were part of the primary intention-to-treat analysis. Table 2 shows the results of analyses on the primary outcome, on mortality and for subgroups regarding the reduction of antidepressant subclasses. Compared to the control group, the intervention arm had an unadjusted OR of 2.12 for a reduction of total antidepressants (95% CI = 0.77–5.81). After adjusting for the predefined variables, the OR was 1.88 (95% CI = 0.71–4.98). At the end of the intervention, 46 participants had died (22 (35%) in the intervention vs. 24 (36%) in the control). The OR for mortality was 0.96 (0.45–2.04) in the intervention vs. the control group.

**Table 2:** Primary and secondary outcomes

Total number of reductions			Odds ratio (95% CI)	
	***Intervention*** ***(n* = *62)***	***Control*** ***(n* = *66)***	***Unadjusted***	***Adjusted***^***a***^
**Primary outcome**				
Reduction of antidepressants, no. (%)	17 (27)	10 (15)	2.12 (0.77–5.81)	1.88 (0.71–4.98)
**Secondary outcomes**				
Mortality, no.(%)	22 (35)	24 (36)	0.96 (0.45–2.04)	0.83 (0.46–1.51)
**Secondary outcomes;** **Reduction of each class of antidepressants (number of prescriptions)**				
	**No. of reductions (% of baseline prescription for each class)**	**No. of reductions (% of baseline prescription for each class)**	***Unadjusted***	***Adjusted***^***a***^
SSRI (N06 AB…), no.(%)	11 (37) *(n* = *30)*^*b*^	11 (31) *(n* = *36)*^*b*^	1.32 (0.42–4.12)	1.48 (0.42–5.27)
NaSSA(N06 AX11 + N06 AX03), no.(%)	13 (38) *(n* = *34)*^*b*^	6 (22) *(n* = *27b*^*2*^	2.17 (0.48–9.72)	2.20 (0.24–19.97)
SNRI (N06 AX16 + N06 AX21), no.(%)	3 (50) *(n* = *6)*^*b*^	0 (0) *(n* = *7)*^*b*^	N/A^d^	N/A^d^
TCA (N06 AA…), no.(%)	0 (0) *(n* = *1)*^*b*^	0 (0) *(n* = *1)*^*b*^	N/A^d^	N/A^d^
Melatonin agonist (N06 AX22), no.(%)	1 (100) *(n* = *1)*^*b*^	0 (0) *(n* = *1)*^*b*^	N/A^d^	N/A^d^
Serotonin modulating drugs (N06 AX26), no.(%)	N/A^c^	0 (0) *(n* = *1)*^*b*^	N/A^d^	N/A^d^

*SSRI* Selective Serotonin Reuptake Inhibitor, *NaSSA* noradrenergic and specific serotonergic antidepressant, *SNRI* Serotonin and norepinephrine reuptake inhibitors, *TCA* Tricyclic antidepressant

^a^Adjusted for years of practice per GP, time participant had lived in a nursing home, the severity of dementia, the total amount of prescriptions per participant, time on the current antidepressant, receiving antipsychotic or hypnotic or sedative

^b^number of prescriptions at baseline for each antidepressant class

^c^no participant received this class at baseline

^d^ cannot be calculated due to too few observations

It was only possible to report the OR for SSRI and NaSSA since too few participants received the other classes of antidepressants. For SSRI, the OR for a reduction was 1.32 95% CI = 0.42–4.12, and for NaSSA, 2.17 (95% CI = 0.48–9.72).

The remaining secondary outcomes are shown in Table 3. Only antidementia medication had a statistically significant difference in favor of the intervention group but not for the remaining psychotropic medications.

**Table 3:** Secondary outcomes

			**Odds ratio (95% CI)**	
	***Intervention*** ***(n* = *62)***	***Control*** ***(n* = *66)***	***Unadjusted***	***Adjusted***
**Secondary outcomes;** **Reduction of other psychotropic medication (number of prescriptions)**				
	**No. of reductions (% of baseline prescription for each drug)**	**No. of reductions (% of baseline prescription for each drug)**	***Unadjusted***	***Adjusted***^***a***^
Antidementia, no.(%)	5 (36) *(n* = *14)*^*b*^	4 (14) *(n* = *29)*^*b*^	3.47 (1.09–11.04)	N/A
Anticonvulsant, no.(%)	3 (43) *(n* = *7)*^*b*^	1 (33) *(n* = *3)*^*b*^	1.50 (0.12–19.44)	N/A
Antipsychotic, no.(%)	2 (29) *(n* = *7)*^*b*^	1 (14) *(n* = *7)*^*b*^	2.40 (0.14–39.93)	N/A
Analgesic, no.(%)	10 (36) *(n* = *28)*^*b*^	11 (33) *(n* = *33)*^*b*^	1.11 (0.39–3.16)	N/A
Anxiolytic, no.(%)	3 (60) *(n* = *5)*^*b*^	4 (80) *(n* = *5)*^*b*^	0.38 (0.04–3.23)	N/A
Sedative or hypnotic, no.(%)	4 (40) *(n* = *10)*^*b*^	3 (23) *(n* = *13)*^*b*^	2.22 (0.37–13.24)	N/A
**Secondary outcomes;** **Hospitalization**				
	***Intervention*** ***(n* = *32)***^***c***^	***Control*** ***(n* = *20)***^***c***^	***Unadjusted with imputation (n* = *128)***	***Adjusted***^***a***^
Any reason, no.(%)	8 (25)	6 (30)	0.73 (0.18–2.90)	N/A
Due to fall, no.(%)	3 (9)	2 (10)	0.70 (0.16–3.00)	N/A
**Secondary outcomes;** **Modified NPI scores**				
	***Intervention*** ***(n* = *15)***^***c***^	***Control*** ***(n* = *10)***^***c***^	***Unadjusted with imputation (n* = *128)***	***Adjusted***^***a***^
Change in modified NPI scores^d^, no.(%)	8 (52)^e^	1 (10)	9.74 (0.64–147.63)	N/A

^a^Not possible to adjust due to too few observations

^b^number of prescriptions at baseline for each class of psychotropic

^c^number of participants with reported data

^d^a change is defined as higher modified NPI score than baseline

^e^four of these were reported in participants with reductions

We had a high proportion of missing data for hospitalization outcomes including falls (59%) and modified NPI scores (50%). After multiple imputations, the OR was 0.73 (95% CI = 0.18–2.90) for hospitalization and 0.70 (95% CI = 0.16–3.00) for falls. The OR for change in modified NPI score was 9.74 (95% CI = 0.64–147.63).

## Discussion

In this trial, we evaluated a three-step intervention designed to assist nursing home GPs in collaboration with nursing home staff, patient and relatives to deprescribe antidepressants in people with dementia living in nursing homes. The intervention aimed to 1) improve the overall knowledge concerning antidepressants and deprescribing for this population (case-based training course), 2) help prepare and structure the observations and thoughts from the nursing home staff (pre-visit reflection tool) and 3) aid and structure the consultation with the nursing home GP concerning antidepressants and possible deprescribing (dialog tool at a structured consultation). Overall, there was a tendency towards a higher deprescribing when using the intervention. However, we failed to show a significant difference in the reduction of antidepressants for participants attending a nursing home GP in the intervention group versus those attending a nursing home GP in the control group. For the secondary outcomes of mortality, reduction of other psychotropic medications, hospitalization and falls, or modified NPI score we only identified a statistically significant effect on the reduction of antidementia medication while the rest was non-significant.

Although there was no difference between the groups, there was an overall tendency towards an increase in reduction in the intervention group compared with the control group when comparing all outcomes. This suggest that there may have been some effect of the intervention. This is consistent with other trials evaluating complex interventions in nursing homes attempting to deprescribe psychotropic medication where mixed results are shown [32, 41–46]. The trials with no effect on the reduction of psychotropic medication included educational material combined with regular medication reviews and the involvement of relatives [32, 45]. These studies also found a large drop-out rate from GPs and inconsistencies in adherence to the intervention [32, 41]. For those trials that managed to show an effect on reduction, there was a combination of more intensive educational sessions, inclusion of “champions” and discussions with colleagues for the medication reviews [43, 44]. A Norwegian study which closely resembled our study, reported a reduction of all psychotropic medication in both intervention and control groups, with the highest number of reductions for antidepressants and sedatives [42]. This study did not show an increase in BPSD symptoms, suggesting that the non-significant increase in BPSD we found is properly due the high rate of missing data or chance, although some caution should be warranted since the increase occurred in four participants in the intervention group with a reduction of antidepressants. In line with the Norwegian study, we also observed a reduction in both groups, suggesting, the enhanced care as usual in out trial may have had an effect [42].

### Strengths and limitations

A strength of the study was the study design, which made it possible to include persons with severe dementia, a population often excluded from trials due to difficulties in obtaining written informed consent [47].

Our study also had several limitations. First of all, the trial was underpowered, due to difficulty of recruiting to sample, including substantial attrition of GPs in both the intervention (two GPs) and control group (three GPs), which is not uncommon in trials within primary care [48]. Due to funding related to the compensation of the GP which was targeted to GPs in the Capital Region and set time of the intervention, it was not possible to extend the inclusion period. Furthermore, the GPs could not include the predefined participants since they did not have the number of patients on antidepressants at their nursing home. Some of the attrition may be due to a work overload on the GPs following COVID-19, and some may be due to time restraints or other issues [49, 50]. Thus, our failure to demonstrate effectiveness may be due to a lack of power. Additionally the difference between the two groups in the proportion of people with a confirmed diagnosis of dementia and suspected diagnosis of dementia may have affected the results. Physicians may be less inclined to deprescribe antidepressants if there is a diagnosis of dementia and BPSD or if the suspected dementia is mild. On the contrary, in Danish nursing homes people with suspected dementia but without an official diagnosis often do not have the diagnosis because they cannot corporate to the full medical examination program required. They often have moderate or severe symptoms which could also influence the deprescribing process.

A complex intervention always carries a risk of not identifying the active component, thus making it difficult to replicate and generalize. However, we conducted a process evaluation that provided insight into the active components [51]. The study faced potential bias issues, since blinding the participants is not possible in behavioral interventions such as this trial. Furthermore the GPs acted both as interventionists and data collectors, which could influence the results. This bias was diminished to a certain extent, since the GPs were blinded to the allocation while collecting baseline data. Additionally the data used for most outcome was printed directly from the medical record. Conceivably, lack of blinding could have given bias in the analyses pertaining the NPI-score; notably, the NPI-score is the outcome with the most missing data. We also acknowledge the major limitation that we had to change the trial period for evaluating the primary outcome stated in the protocol due to missing data. This may have led to a misinterpretation of the results since we cannot be sure how and when the structured consultations performed in the intervention group occurred and if the reduction was caused by this. However, when comparing our results to available literature, our findings are in line with those studies which suggest that the change of the period most likely did affect the results. Compliance is hard to assess since it involves the adherence to certain guidelines and the use of a tool; in many cases it is unclear whether the patient and the doctor used the tool as this was rarely recorded. Hence, the lack of effect may be because of non-compliance, i.e. maybe many doctors in the intervention group just ignored the tool, but we cannot gauge how many. Additionally, a large proportion of missing data for the secondary analysis occurred, which may cause an underestimation of the potential harm of the intervention according to the modified NPI and hospitalization. Since we had very few who reported modified NPI scores, the risk that specific reasons for the staff to not report symptoms of the participants could have influenced the results. Although electronic medical charts provided most of the data, missing data for many of especially secondary outcomes still occurred. This is often an issue with intervention research on older people, since there is a natural, high mortality rate. For future research it is important to consider more appropriate ways to ensure more complete data collection.

It may also be that despite our efforts to customize the intervention to the nursing home setting, there may still be issues with the intervention that we did not fully understand. Additionally, the tailored intervention and the possibility of the GPs to further customize the intervention, have decreased the generalizability of the study.

## Conclusion

A combined intervention, including a training session with case-based material, a pre-reflection tool, and a structured consultation with the use of a dialog tool, was not effective in reducing the use of antidepressants in nursing home residents with dementia. The intervention group had more reductions of antidepressants and other drugs during the intervention period than the control group, but the differences were mostly not significant. For future research, it would be valuable to further optimize the intervention before testing it in a more well-powered study to elaborate on potential beneficial effects and whether there are potential harms when attempting to deprescribe antidepressants in this population.

## Supplementary Information

Supplementary Material 1. CONSORT Checklist.

## Data Availability

Data may be made available through formal data sharing agreement with the authors’ institution on reasonable request.

## Abbreviations

ATC: Anatomical Therapeutic Chemical
BPSD: Behavioral and Psychological Symptoms of Dementia
CONSORT: Consolidated Standards of Reporting Trials
cRCT: Cluster Randomized Controlled Trial
DDD: Defined Daily Dose
GEE: Generalized Estimating Equation
GP: General Practitioners
MMSE: Mini Mental State Examination
MRC: Medical Research Council
NaSSA: Noradrenergic and Specific Serotonergic Antidepressant
NPI: NeuroPsychiatric Inventory
NPI-NH: NeuroPsychiatric Inventory, Nursing Home edition
OR: Odds Ratio
SNRI: Serotonin and Norepinephrine Reuptake Inhibitors
SSRI: Selective Serotonin Reuptake Inhibitor
TCA: TriCyclic Antidepressant
